# Effects of Acute and Chronic Gamma Irradiation on the Cell Biology and Physiology of Rice Plants

**DOI:** 10.3390/plants10030439

**Published:** 2021-02-25

**Authors:** Hong-Il Choi, Sung Min Han, Yeong Deuk Jo, Min Jeong Hong, Sang Hoon Kim, Jin-Baek Kim

**Affiliations:** 1 Advanced Radiation Technology Institute, Korea Atomic Energy Research Institute, Jeongeup 56212, Korea; hichoi@kaeri.re.kr (H.-I.C.); jyd@kaeri.re.kr (Y.D.J.); hongmj@kaeri.re.kr (M.J.H.); shkim80@kaeri.re.kr (S.H.K.); 2 Division of Ecological Safety, National Institute of Ecology, Seocheon 33657, Korea; smhan@nie.re.kr

**Keywords:** acute and chronic irradiation, DNA damage, oxidative stress, antioxidant activity, photosynthetic efficiency, growth and reproduction

## Abstract

The response to gamma irradiation varies among plant species and is affected by the total irradiation dose and dose rate. In this study, we examined the immediate and ensuing responses to acute and chronic gamma irradiation in rice (*Oryza sativa* L.). Rice plants at the tillering stage were exposed to gamma rays for 8 h (acute irradiation) or 10 days (chronic irradiation), with a total irradiation dose of 100, 200, or 300 Gy. Plants exposed to gamma irradiation were then analyzed for DNA damage, oxidative stress indicators including free radical content and lipid peroxidation, radical scavenging, and antioxidant activity. The results showed that all stress indices increased immediately after exposure to both acute and chronic irradiation in a dose-dependent manner, and acute irradiation had a greater effect on plants than chronic irradiation. The photosynthetic efficiency and growth of plants measured at 10, 20, and 30 days post-irradiation decreased in irradiated plants, i.e., these two parameters were more severely affected by acute irradiation than by chronic irradiation. In contrast, acutely irradiated plants produced seeds with dramatically decreased fertility rate, and chronically irradiated plants failed to produce fertile seeds, i.e., reproduction was more severely affected by chronic irradiation than by acute irradiation. Overall, our findings suggest that acute gamma irradiation causes instantaneous and greater damage to plant physiology, whereas chronic gamma irradiation causes long-term damage, leading to reproductive failure.

## 1. Introduction

Ionizing radiation is a primeval stressor from an evolutionary point of view, as all life on Earth has evolved in the presence of various naturally occurring background radiation sources of both cosmic and geologic origin [1]. Gamma rays are an extremely penetrating form of electromagnetic radiation arising from the nuclei of radioactive atoms, which were first observed by Paul Villard in 1900 and named by Ernest Rutherford in 1903 [2,3]. To generate artificial gamma rays, the irradiation facilities use radionuclide sources, such as ^60^Co, which emit gamma rays at a dose rate that is proportional to the activity of the radioactive source and inversely proportional to the square of the distance from the radioactive source [4]. Even if an equal dose is irradiated, the irradiation time varies with the dose rate, ranging from hours with high dose rates to weeks with low dose rates. In general, high dose rates cause acute irradiation, whereas low dose rates lead to chronic irradiation [1,5].

Exposure to ionizing radiation causes direct or indirect damage in plants. Direct damage occurs when the radiation energy is transferred to cells and DNA directly, leading to cell damage or cell death and inducing abnormalities [6,7]. Indirect damage is caused by reactive oxygen species (ROS) such as hydroxyl radical (^•^OH), hydrogen peroxide (H_2_O_2_), and superoxide anion (O_2_^•−^), which are generated by water radiolysis and lead to oxidative stress [6,7,8]. The hydroxyl radical is highly reactive and can rapidly oxidize macromolecules in cells, causing lipid peroxidation, protein oxidation, and DNA damage [6,8]. DNA damage is known to be induced more by ROS than by direct irradiation [6,9]. Under oxidative stress conditions, plants activate their own antioxidant defense systems to scavenge ROS. Plants employ two kinds of antioxidant machineries: (i) enzymatic machinery comprising antioxidant enzymes such as ascorbate peroxidase (APX), catalase (CAT), peroxidase (POD), and superoxide dismutase (SOD); and (ii) non-enzymatic machinery, which includes antioxidant metabolites such as ascorbate and glutathione or phytochemicals such as anthocyanins, carotenoids, and phenolic compounds [8,10].

According to the theory of radiation hormesis, gamma irradiation at a low dose has a positive effect on plant growth [11]. However, exposure to high doses of gamma irradiation above a certain threshold has negative effects on plant growth and development [12]. The reasons why gamma irradiation inhibits plant growth and development are complicated by the direct and indirect effects of radiation, among which reduction in photosynthetic capacity is considered one of the most crucial factors [10,13,14]. Experimental evidence suggests that chloroplast is vulnerable to high-dose gamma irradiation, as it leads to the deformation of the thylakoid structure, thereby reducing photosynthetic efficiency [15,16]. Because the energy required for completing the plant life cycle is obtained through photosynthesis, ionizing radiation-induced decline in photosynthetic efficiency has harmful effects on plant growth, development, and reproduction. Above all, reproduction is greatly influenced by radiation because the production of seeds requires proper division of cells through mitosis to meiosis in specific tissues and at specific times [1,14].

In this study, we irradiated rice plants at the tillering stage with 100, 200, and 300 Gy of gamma rays for two different durations: 8 h (acute irradiation; hereafter referred to as A-100, A-200, and A-300 treatments, respectively) and 10 days (chronic irradiation; hereafter referred to as C-100, C-200, and C-300 treatments, respectively). Then, we compared the effects of acute and chronic irradiation on plants in terms of DNA damage, oxidative stress, antioxidant enzyme activity, photosynthetic efficiency, growth, and reproduction. The results obtained in this study demonstrate the different biological responses of plants to oxidative stress induced by acute and chronic irradiation in rice and the survival strategies employed by plants to overcome this stress.

## 2. Results

### 2.1. DNA Damage

**Figure 1:**
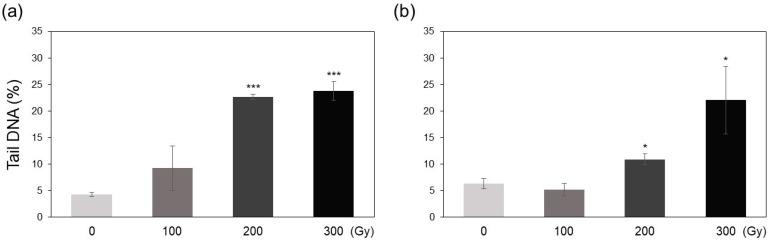
DNA damage induced by acute and chronic gamma irradiation in rice plants. (**a**) Acute irradiation treatments; (**b**) chronic irradiation treatments. Data represent mean ± standard deviation (SD). Asterisks indicate significant differences between control (0 Gy) and other treatments (* *p* < 0.05, *** *p* < 0.001).

DNA damage in irradiated plants was estimated using the comet assay, which quantifies DNA damage by measuring the percentage of DNA in the comet tail observed using single-cell gel electrophoresis [17]. The amount of tail DNA was significantly higher at high doses (200 and 300 Gy) in both acute and chronic irradiation treatments than at low doses (0 and 100 Gy) (Figure 1). The average tail DNA value in A-200 treatment was 22.7%, which was similar to that observed in A-300 (23.8%) and C-300 (22.1%) treatments (Figure 1). The amount of DNA damage was higher in acute irradiation treatments than in chronic treatments; however, neither A-100 nor C-100 showed significant difference compared with the non-irradiated controls (Figure 1).

### 2.2. Free Radicals, H_2_O_2_, and Lipid Peroxidation

**Figure 2:**
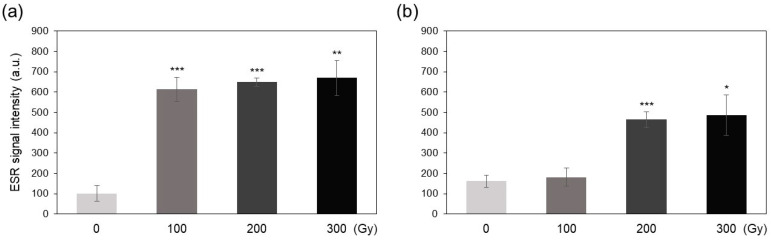
Determination of the free radical content of gamma-irradiated rice plants using the electron spin resonance (ESR) method. (**a**,**b**) ESR intensity in rice plants subjected to acute (**a**) and chronic (**b**) gamma irradiation. Data represent mean ± SD. Asterisks indicate significant differences between control (0 Gy) and other treatments (* *p* < 0.05, ** *p* < 0.01, *** *p* < 0.001).

The electron spin resonance (ESR) method was employed to quantify free radicals produced in rice plants by acute and chronic irradiation. The intensity of ESR signals, which corresponds to the free radical content, increased sharply in the acutely irradiated plants to levels 6-fold higher than that in non-irradiated control plants (Figure 2a). Chronic irradiation at 200 and 300 Gy also increased the free radical content to approximately 3-fold higher levels compared with that of the control, whereas the C-100 treatment showed no significant increase in the free radical content (Figure 2b).

**Figure 3:**
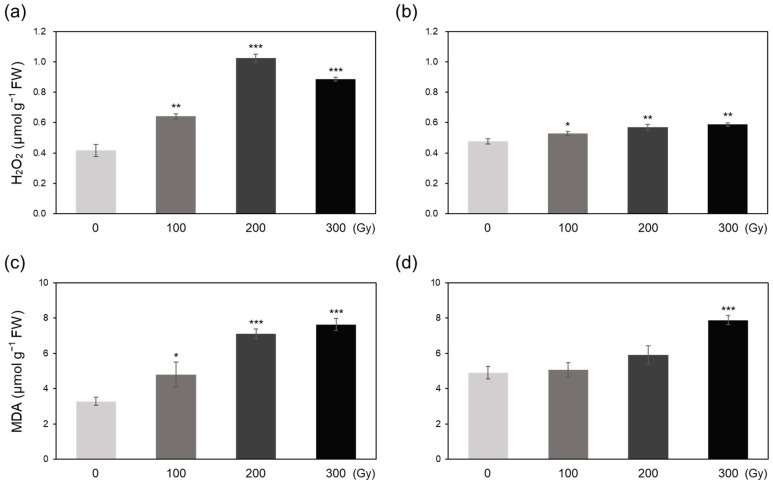
Quantification of hydrogen peroxide (H_2_O_2_) and malondialdehyde (MDA) contents of rice plants subjected to acute and chronic gamma irradiation. (**a**,**b**) H_2_O_2_ contents of plants subjected to acute (**a**) and chronic (**b**) gamma irradiation. (**c**,**d**) MDA contents of plants subjected to acute (**c**) and chronic (**d**) gamma irradiation. Data represent mean ± SD. Asterisks indicate significant differences between control (0 Gy) and other treatments (* *p* < 0.05, ** *p* < 0.01, *** *p* < 0.001).

The H_2_O_2_ content of rice plants significantly increased upon both acute and chronic irradiation (Figure 3a,b). Overall increase in the H_2_O_2_ level was larger in the acutely irradiated plants than in the chronically irradiated plants and its content in A-200 and A-300 was more than doubled compared with that in their control (Figure 3a). Chronic irradiation gradually increased the H_2_O_2_ content by 10.7% (C-100) to 23.3% (C-300) (Figure 3b). Malondialdehyde (MDA) content also increased significantly upon acute irradiation in a dose-dependent manner (Figure 3c,d). The MDA content of plants in A-200 and A-300 treatments was >2-fold higher than that of control plants (Figure 3c). A small, but non-significant, increase in MDA content was observed in C-100 and C-200 treatments, whereas the content increased by 60.5% in C-300 (Figure 3d).

### 2.3. Superoxide Radical Scavenging and Antioxidant Activities

**Figure 4:**
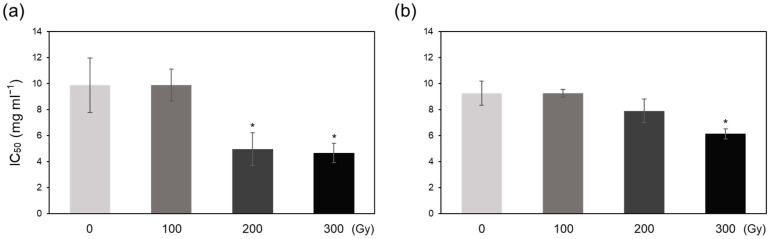
Estimation of superoxide radical (O_2_^•−^) scavenging activity in gamma-irradiated rice plants by measuring half maximal inhibitory concentration (IC_50_) values. (**a**,**b**) IC_50_ values of plants subjected to acute (**a**) and chronic (**b**) gamma irradiation. Data represent mean ± SD. Asterisks indicate significant differences between control (0 Gy) and other treatments (* *p* < 0.05).

Compared with the control, A-100 and C-100 showed slightly lower half-maximal inhibitory concentration (IC_50_) of O_2_^•−^, whereas A-200, A-300, and C-300 treatments showed significantly decreased IC_50_ values (Figure 4). C-200 also showed a small reduction in IC_50_ value compared with the control, but the difference was not significant (Figure 4b). These results indicate that high-dose acute and chronic irradiation increases radical scavenging activity.

**Figure 5:**
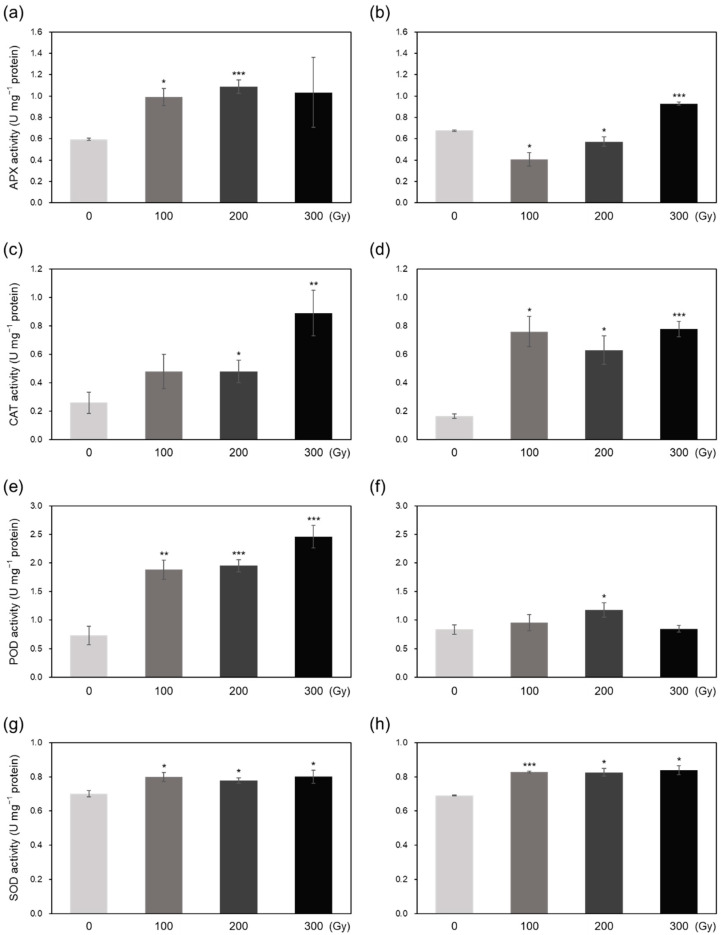
Measurement of antioxidant enzyme activities in rice plants. (**a**–**h**) Activities of ascorbate peroxidase (APX) (**a**,**b**), catalase (CAT) (**c**,**d**), peroxidase (POD) (**e**,**f**), and superoxide dismutase (SOD) (**g**,**h**) in plants subjected to acute (**a**,**c**,**e**,**g**) and chronic (**b**,**d**,**f**,**h**) gamma irradiation. Data represent mean ± SD. Asterisks indicate significant differences between the control (0 Gy) and other treatments (* *p* < 0.05, ** *p* < 0.01, *** *p* < 0.001).

The activity of four antioxidant enzymes generally increased in the both acutely and chronically irradiated plants (Figure 5). Compared with the control, APX activity increased by at least 2-fold in acute irradiation treatments; however, among chronic irradiation treatments, APX activity was lower in C-100 and C-200 and higher in C-300 compared with that in the control (Figure 5a,b). CAT activity notably increased upon both acute and chronic irradiation, although CAT activity was 1.8- to 3.4-fold higher in acute irradiation treatments and 3.8- to 4.7-fold higher in chronic irradiation treatments compared with that in the control (Figure 5c,d). The activity of POD was 2.6- to 3.4-fold higher in acutely irradiated plants compared with that in the control; however, among chronic irradiation treatments, only C-200 showed significantly higher POD activity than the control (Figure 5e,f). SOD activity was increased slightly by both acute and chronic irradiation (Figure 5g,h).

**Figure 6:**
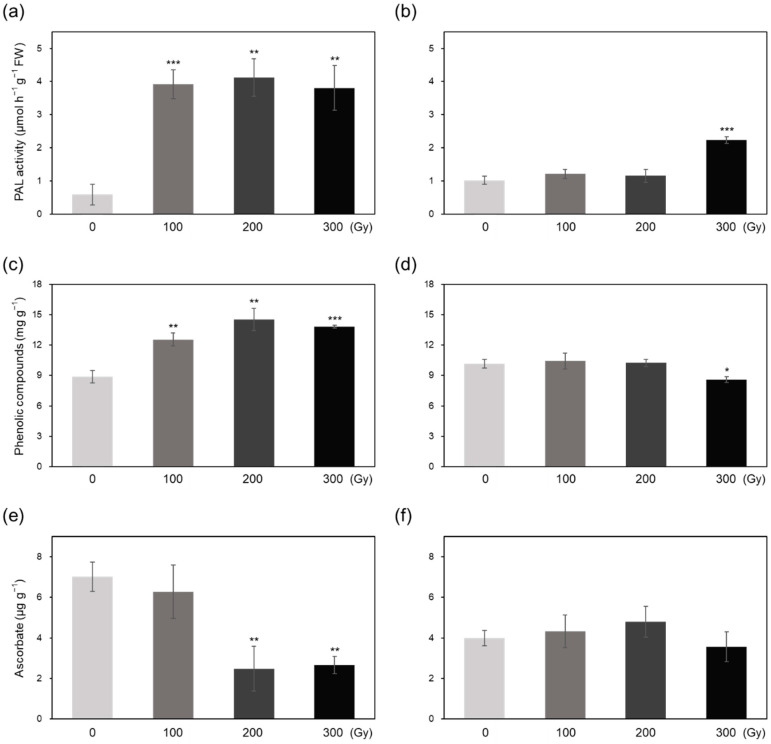
Phenylalanine ammonia-lyase (PAL) activity, phenolic compound content, and ascorbic acid (AsA) content of rice plants subjected to gamma irradiation. (**a**–**f**) PAL activity (**a**,**b**), phenolic compound content (**c**,**d**), and AsA content (**e**,**f**) of rice plants subjected to acute (**a**,**c**,**e**) and chronic (**b**,**d**,**f**) gamma irradiation. Data represent mean ± SD. Asterisks indicate significant differences between control (0 Gy) and other treatments (* *p* < 0.05, ** *p* < 0.01, *** *p* < 0.001).

Phenylalanine ammonia-lyase (PAL), a key enzyme in the phenylpropanoid biosynthesis pathway, showed higher activity in both acutely and chronically irradiated plants compared with that in the control (Figure 6a,b). Acute irradiation induced an approximately 7-fold increase in PAL activity at all three doses, whereas chronic irradiation induced a 2-fold increase in PAL activity at 300 Gy compared with the control (Figure 6a,b). Additionally, the phenolic content of rice plants increased by 1.5-fold upon acute irradiation, but either showed an insignificant increase (C-100 and C-200) or decreased (C-300) upon chronic irradiation (Figure 6c,d). The content of another non-enzymatic antioxidant, ascorbic acid (AsA), decreased significantly in A-200 and A-300 treatments by 65% but showed no significant change in chronically irradiated plants (Figure 6e,f).

### 2.4. Photosynthesis Efficiency

**Figure 7:**
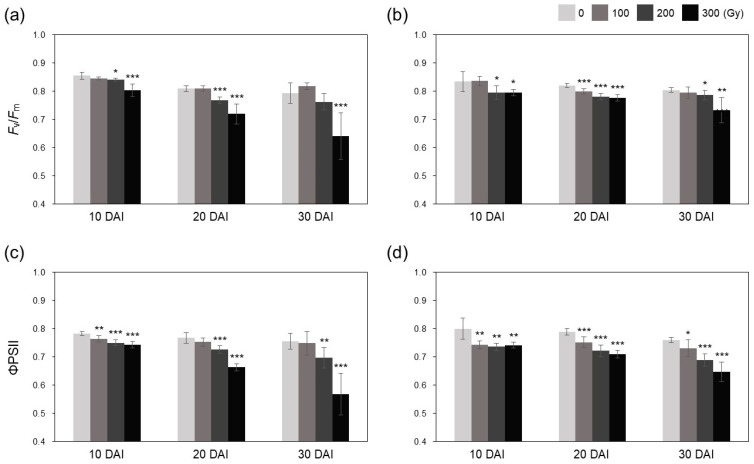
Photosynthetic efficiency of rice plants subjected to gamma irradiation. (**a**–**d**) Maximum quantum yield of PSII (*F*_v_/*F*_m_) (**a**,**b**) and quantum yield of PSII electron transport (ΦPSII) (**c**,**d**) in rice plants subjected to acute (**a**,**c**) and chronic (**b**,**d**) gamma irradiation. DAI, days after irradiation. Data represent mean ± SD. Asterisks indicate significant differences between control (0 Gy) and other treatments (* *p* < 0.05, ** *p* < 0.01, *** *p* < 0.001).

To determine the effect of gamma irradiation on the photosynthetic efficiency of rice plants, we measured the maximum quantum yield of PSII (*F*_v_/*F*_m_) and quantum yield of PSII electron transport (ΦPSII) at 10, 30, and 50 days after irradiation (DAI). Both *F*_v_/*F*_m_ and ΦPSII decreased in irradiated plants compared with those in non-irradiated control plants (Figure 7). The difference in *F*_v_/*F*_m_ and ΦPSII values between the control and irradiated plants increased over time in a dose-dependent manner, and this difference was greater in acute irradiation treatments than in chronic treatments. In the A-300 treatment, *F*_v_/*F*_m_ decreased by 11.1% at 30 DAI and by 19.1% at 50 DAI, whereas ΦPSII decreased by 13.5% at 30 DAI and by 24.9% at 50 DAI (Figure 7c). The C-300 treatment showed 5.3% and 8.8% decrease in *F*_v_/*F*_m_ and 10.0% and 14.7% decrease in ΦPSII at 30 DAI and 50 DAI, respectively, compared with the control (Figure 7d). These results indicate that the cumulative oxidative stress induced by acute irradiation was greater than that induced by chronic irradiation.

### 2.5. Growth and Reproduction

**Figure 8:**
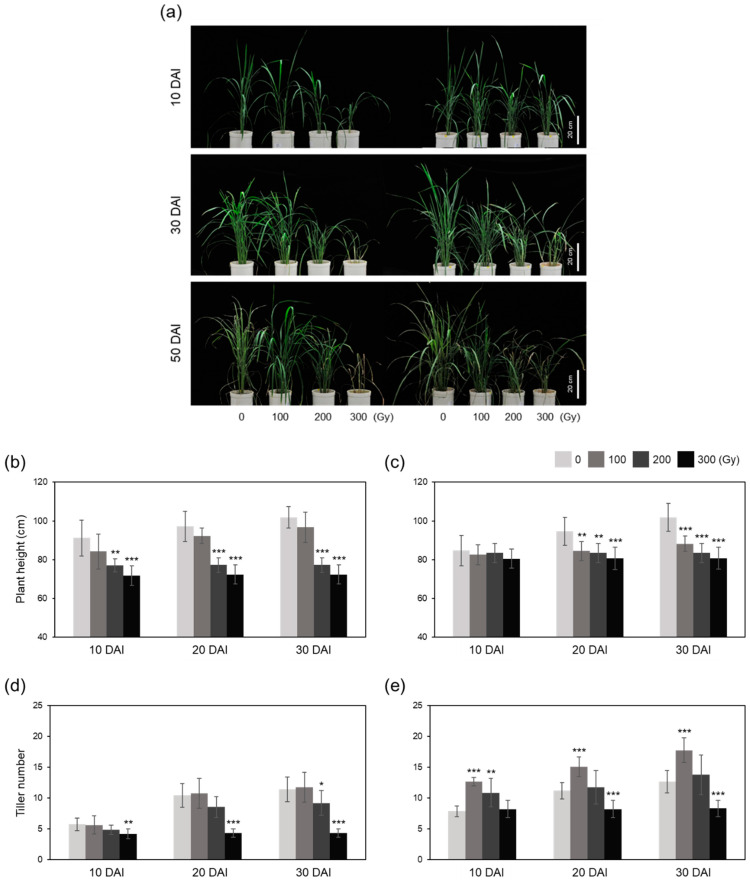
Growth analysis of rice plants exposed to gamma irradiation. (**a**) Photographs of rice plants treated with acute and chronic gamma irradiation. (**b**–**e**) Plant height (**b**,**c**) and tiller number (**d**,**e**) of rice plants subjected to acute (**b**,**d**) and chronic (**c**,**e**) gamma irradiation. DAI, days after irradiation. Data represent mean ± SD. Asterisks indicate significant differences between control (0 Gy) and other treatments (* *p* < 0.05, ** *p* < 0.01, *** *p* < 0.001).

Plant growth parameters were measured at 10, 30, and 50 DAI. The increase in plant height was inhibited at 100 Gy and almost stopped at 200 and 300 Gy gamma rays in both acute and chronic irradiation treatments (Figure 8a–c). However, acute and chronic irradiation showed different effects on tiller number (Figure 8a,d,e). The A-100 and A-200 treatments showed similar tiller numbers, which were low and similar to the control at all-time points; however, the A-300 treatment caused the plants to stop growing (Figure 8d). However, the tiller numbers were significantly higher in C-100 and C-200 treatments at 10 DAI compared with those in the control (Figure 8e). Additionally, the number of tillers in C-100 at 30 and 50 DAI was 34.7% and 40.4% higher than that of the control, respectively (Figure 8e). The increase in tiller number in C-200 slowed down at 30 and 50 DAI, whereas tiller growth stopped in C-300, similar to A-300 (Figure 8e).

We also surveyed the panicle number (PN), panicle length (PL), spikelet number per panicle (SN), and fertility rate (FR) of rice plants to evaluate the effects of gamma irradiation on reproduction in rice plants. The results showed that both acute and chronic irradiation negatively affected plant reproduction (Table 1). The A-100 treatment showed a slight reduction in PN (4.1) and PL (17.1 cm) but a distinct reduction in SN (63.6) and FR (42.6%) compared with the control (4.6, 18.4 cm, 98.6, and 71.1%, respectively) (Table 1). The A-200 treatment showed a drastic decrease in PN (2.0), PL (11.3 cm), SN (24.4), and FR (5.0%) (Table 1). In the A-300 treatment, plants withered and died before reaching the heading stage (Table 1). Among the chronic irradiation treatments, only C-100 plants produced panicles, whereas C-200 and C-300 plants died before producing panicles, similar to A-300 plants (Table 1). However, the values of PN, PL, and SN in C-100 plants were slightly lower compared with those in the control, and these plants did not produce any fertile seed (Table 1).

## 3. Discussion

This study aimed to determine the differences in biological effects of acute and chronic gamma irradiation on rice plants. Immediately after irradiation, the direct and indirect indicators of irradiation-induced damage including DNA degradation, free radical content, and MDA content increased in a dose-dependent manner, and the increase was generally greater in the acutely irradiated plants than in chronically irradiated plants (Figure 1, Figure 2 and Figure 3). These results suggest that high dose rates induce stronger damage compared with low dose rates of chronic irradiation. It is generally acknowledged that higher irradiation doses lead to more severe biological damage [10,16,18]; thus, the results of the current study are consistent with those of previous studies.

Irradiation induces oxidative stress via excessive ROS production, which activates enzymatic and non-enzymatic antioxidant defense systems in plants to induce ROS scavenging [6,19]. Low IC_50_ values observed in high-dose irradiated plants (Figure 4) demonstrates that higher doses activate the antioxidant defense systems to a greater extent than lower doses in both acute and chronic irradiation treatments to mitigate the detrimental effects of ROS [8,20]. Generally, antioxidant enzyme activities showed a greater increase in acutely irradiated plants than in chronically irradiated plants, implying a more prompt response of enzymatic ROS scavenging to irradiation at a high dose rate (Figure 5). Exceptionally, CAT activity increased more by chronic irradiation than by acute irradiation (Figure 5c,d). In that CAT catalyzes the reaction to decompose H_2_O_2_ into H_2_O and O_2_ [8], the high CAT activity in chronic irradiation treatment may contribute to preventing an excessive increase in H_2_O_2_ content (Figure 3b). PAL catalyzes the first step in the phenylpropanoid pathway, i.e., the conversion of L-phenylalanine into *trans*-cinnamate, which plays an important role in the production of antioxidant phenolic compounds such as flavonoids and tannins [21,22]. Gamma irradiation has been shown to increase PAL activity in peach [23]. In this study, PAL activity increased dramatically upon acute irradiation, which may be related to the high-level accumulation of phenolic compounds (Figure 6a,c). The decrease in the AsA content of acutely irradiated plants was likely induced by its consumption for ROS scavenging. Taken together, these data suggest that antioxidant defense systems are more active immediately after acute irradiation than after chronic irradiation, and different dose rates cause different ROS scavenging responses between acute and chronic irradiation treatments.

Photosynthesis is a carbon fixation process essential for plant growth and survival [24]. High-dose irradiation hinders photosynthesis by inhibiting the biosynthesis and degradation of chlorophyll [10,13,16]. In this study, the photosynthetic efficiency of rice plants was reduced by gamma irradiation over time in a dose-dependent manner, and this reduction was greater in acutely irradiated plants than in chronically irradiated plants (Figure 7). This indicates that high-dose irradiation causes severe physiological damage that cannot be recovered over time, and damage caused by high dose rate is more irreparable than that caused by low dose rate. This tendency was also revealed in the vegetative growth test; both A-300 and C-300 treatments almost stopped plant growth, and A-100, A-200, C-100, and C-200 treatments inhibited the increase in plant height, although C-100 and C-200 treatments significantly increased the tiller number compared with the control (Figure 8).

Reproduction is one of the most intrinsic and indispensable characteristics of all living organisms and is required for the perpetuation of any given species [25]. Unlike the results of all other assays, reproductive damage was greater in chronically irradiated plants than in acutely irradiated plants, and the C-100 treatment resulted in the failure to produce seeds (Table 1). Although A-100 and A-200 produced mature seeds, plant reproduction was hindered in these treatments (Table 1). This can be explained by the cost of reproduction hypothesis, the trade-offs between reproduction and life-history traits [26,27]. Plants irradiated at a low dose rate may activate antioxidant systems to overcome oxidative stress during and after the chronic irradiation, spanning a large part of the vegetative growth phase, however, presumably resulting in reproductive failure because of their efforts to deal with oxidative stress in vegetative period. In addition, the end point of chronic irradiation is much closer to the heading stage than that of acute irradiation; hence, the damage caused to a single cell, which is about to develop into a gamete, can lead to reproductive failure [14].

In conclusion, this study demonstrates different physiological and biochemical responses of rice plants to acute and chronic irradiation, ranging from responses that occur immediately post-irradiation to those that occur during subsequent vegetative growth and reproduction. In most of the assays, acutely irradiated plants seemed to suffer more damage during the vegetative growth phase, thus activating oxidative stress management systems, whereas chronically irradiated plants suffered more reproductive damage, leading to the inability to produce seeds. Thus, the results of this study enhance our knowledge of how rice plants respond to acute and chronic irradiation and withstand oxidative stress.

## 4. Materials and Methods

### 4.1. Sample Preparation, Growth, and Growth Test after Irradiation

Rice (*Oryza sativa* L. ssp. *japonica* cv. Ilpum) plants grown for 55 days after sowing were subjected to gamma-irradiation. Two gamma irradiation facilities at the Advanced Radiation Technology Institute, Korea Atomic Energy Research Institute (Jeongeup, Republic of Korea) were used in this study. At these facilities, a ^60^Co gamma irradiator (150 TBq; Nordion, Ontario, Canada) was used for 8-h acute irradiation, and a ^60^Co gamma phytotron (20 TBq; Nordion, Ontario, Canada) was used for 10-day chronic irradiation. Final exposure doses were set at 100, 200, and 300 Gy for both acute and chronic irradiation. Therefore, dose rates for A-100, A-200, and A-300 treatments were 12.5, 25.0, and 37.5 Gy/h, respectively, and those for C-100, C-200, and C-300 treatments were 0.417, 0.833, and 1.25 Gy/h, respectively. Non-irradiated control plants were prepared separately because of the differences in exposure time and environmental conditions between acute and chronic irradiation treatments. Each treatment contained 10 plants. Leaf samples for further experimental analysis were collected immediately after irradiation. To measure photosynthetic efficiency, plant growth, and reproductive traits, the control and irradiated plants were grown in a greenhouse. Plant height and tiller number were measured at 10, 30, and 50 DAI. Reproductive indices (PN, PL, SN, and FR) were measured after the heading and ripening stages.

### 4.2. Comet Assay

The alkaline comet assay was performed as described previously [18]. Briefly, the first layer of base slides was prepared with 1% normal melting agarose. Leaf samples harvested immediately after irradiation were sliced with a clean razor blade in 1 mL of phosphate-buffered saline (160 mM NaCl, 8 mM Na_2_HPO_4_, 4 mM NaH_2_PO_4_, 50 mM ethylenediaminetetraacetic acid (EDTA) [pH 7.0]) and incubated on ice for 2 h. A 70-µL suspension of nuclei of each sample was mixed with an equal volume of 1% low melting point agarose (Gibco BRL, Gaithersburg, MD, USA). The mixture was subsequently dropped onto the first layer of the base slides, covered with a coverslip, and then solidified. The slides coated with the nuclei were dipped in chilled high-saline lysing buffer (2.5 M NaCl, 10 mM Trizma base (pH 7.5), 100 mM EDTA) after removing the coverslip and stored at 4 °C for 2 h. The nuclei were unwound in high-alkaline electrophoresis buffer (300 mM NaOH, 1 mM EDTA (pH > 13)) for 10 min and then electrophoresed at 25 V (0.7 V/cm) and 300 mA for 10 min. The slides were washed twice with neutralization buffer (0.4 M Tris–HCl (pH 7.5)) after electrophoresis, dehydrated using cold 100% ethanol for 20 min, and air-dried overnight at 24 °C. The nuclear DNA was stained with propidium iodide and observed under a fluorescence microscope (Axio Scope.A1; Carl Zeiss, Oberkochen, Germany) to visualize the DNA content and DNA damage patterns.

### 4.3. Electron Spin Resonance (ESR) Analysis

Leaf samples collected immediately after irradiation were lyophilized and ground using a mortar and pestle. The ground leaf tissue (500 mg) was placed in ESR quartz tubes and sealed. The ESR spectrum was measured directly using the JES-FA200 spectrometer (JEOL, Tokyo, Japan) under the following conditions: modulation frequency, 100 kHz; center field, 328.0 mT; microwave power, 0.998 mW; microwave frequency, 9.197 GHz; modulation width, 0.06 mT; sweep time, 30 s; and time constant, 0.03 s. The ESR signal intensities were obtained directly from each experimental spectrum, and the peak-to-peak amplitude of the first-derivative spectrum was measured for each sample.

### 4.4. H_2_O_2_ and Malonedialdehyde (MDA) Content

The concentration of H_2_O_2_ was measured using a hydrogen peroxide assay kit (ab102500, Abcam, Cambridge, UK), according to the manufacturer’s instructions. Absorbance values were measured at 570 nm using an Infinite 200 Pro microplate reader (Tecan, Mannendorf, Switzerland) and converted into concentrations based on standard curve data. The level of lipid peroxidation was determined by measuring the MDA content using the thiobarbituric acid test, as described previously [18]. Absorbance values were measured at 450, 532, and 600 nm and converted to concentrations (nM g^−1^ fresh weight) using the following equation:

MDA content = 6.453 (A_532_ − A_600_) − 0.563 (A_450_).

### 4.5. Superoxide Radical Scavenging Activity

The superoxide radical scavenging activity was measured using a BJL ultra-weak chemiluminescence analyzer (UWLA; model BPCL-2-KIC; Jye Horm Co., Taipei, Taiwan). A reaction mixture containing lucigenin, phosphate-buffered saline, arginine, and methylglyoxal was prepared as described previously [28], and the mixture was placed in a quartz round-bottom cuvette. The ultra-weak photon counts were recorded by the UWLA, and the IC_50_ value (the concentration required to inhibit 50% of lucigenin-based chemiluminescence of a sample) was calculated from a concentration-inhibition built.

### 4.6. Antioxidant Enzyme Activity

Leaves were ground to a fine powder in the presence of liquid nitrogen. Total proteins were extracted using 0.2 M potassium phosphate buffer containing 0.1 mM EDTA (pH 7.0), and the protein concentration was measured using the Bradford assay, with bovine serum albumin as a standard [29]. APX activity was determined at 290 nm in a reaction mixture containing 50 mM potassium phosphate buffer (pH 7.0), 0.6 mM ascorbate, and 1 mM H_2_O_2_ [10]; POD activity was determined in a reaction mixture containing 100 mM potassium phosphate buffer (pH 6.0), 0.147 M H_2_O_2_, and 5% pyrogallol at 420 nm [10]; CAT activity was determined at 240 nm in a reaction mixture containing 50 mM potassium phosphate buffer (pH 7.0) and 15 mM H_2_O_2_ [10]; SOD activity was determined at 560 nm in a reaction mixture containing 50 mM potassium phosphate buffer (pH 7.8), 0.1 mM EDTA, 13 mM methionine, 75 µM nitro blue tetrazolium, and 10 µM riboflavin [10]. The absorbance was measured using a UV–Vis spectrophotometer (Model 6505; Jenway, Keison Products, Chelmsford, UK).

### 4.7. Phenylanine Ammonia-Lyase (PAL) Activity, Total Phenolic Content, and Ascorbic Acid (AsA) Content

PAL activity was measured by monitoring the reaction product, *trans*-cinnamate [30]. One unit of PAL activity was defined as the amount responsible for increasing the absorbance of the solution at 290 nm by 0.01 per min. The total phenolic content was determined according to the method of Swain and Hillis [31], which was modified according to the recommendation of Ainsworth and Gillespie [32]. The AsA content was determined using an ascorbic acid assay kit (catalog number: ab65346; Abcam, Cambridge, UK). Absorbance was recorded at 570 nm using a standard 96-well plate reader (Tecan, Mannendorf, Switzerland).

### 4.8. Chlorophyll Fluorescence

Chlorophyll fluorescence was measured using an IMAGING-PAM chlorophyll fluorometer (Walz, Effeltrich, Germany). The maximum quantum yield of PSII was calculated as the ratio of variable fluorescence (*F*_v_) to maximal fluorescence (*F*_m_), as described previously [33]. *Fv* was calculated by subtracting the minimum chlorophyll fluorescence (*F*_o_) from *F*_m_. Readings were taken after the samples were dark-adapted for 20 min at room temperature. The value of ΦPSII was calculated using the following equation [34]:

ΦPSII = (*F*_m_′ − *F*′)/*F*_m_′

where *F*_m_′ and *F*′ represent steady-state levels and the maximum yield of fluorescence in light-acclimated samples reached by the application of a saturation pulse, respectively.

### 4.9. Statistical Analysis

Experiments were performed at least in triplicate. Significant differences between non-irradiated and irradiated plants were estimated using Student’s *t*-test (*p* < 0.05; SAS Institute, Cary, NC, USA).

## Figures and Tables

**Table 1:** Reproduction in rice plants subjected to acute and chronic gamma irradiation.

Mode	Dose (Gy)	PN ^1^	PL (cm) ^2^	SN ^3^	FR (%) ^4^
Acute irradiation	0	4.6 ± 0.5	18.4 ± 1.1	98.6 ± 20.7	71.1 ± 16.3
	100	4.1 ± 1.0	17.1 ± 2.0 **	63.6 ± 21.4 ***	42.6 ± 19.4 ***
	200	2.0 ± 0.8 ***	11.3 ± 2.1 ***	24.4 ± 12.1 ***	5.0 ± 5.5 ***
	300	–	–	–	–
Chronic irradiation	0	3.3 ± 0.8	17.2 ± 1.0	58.2 ± 18.9	86.6 ± 8.6
	100	2.1 ± 0.7 ***	14.9 ± 2.8 **	44.7 ± 20.6	0
	200	–	–	–	–
	300	–	–	–	–

^1^ Panicle number per plant. ^2^ Panicle length. ^3^ Spikelet number per plant. ^4^ Fertility rate (percent filled spikelets relative to the total number of spikelets). ** *p* < 0.01; *** *p* < 0.001.
